# Fava Bean Protein Nanofibrils Modulate Cell Membrane Interfaces for Biomolecular Interactions as Unveiled by Atomic Force Microscopy

**DOI:** 10.3390/foods13213411

**Published:** 2024-10-26

**Authors:** Sanjai Karanth, Marina Wiesenfarth, Julia Benthin, Melanie Koehler

**Affiliations:** 1 Leibniz Institute for Food Systems Biology at the Technical University of Munich, Lise-Meitner-Str. 34, 85354 Freising, Germany; s.karanth.leibniz-lsb@tum.de (S.K.); m.wiesenfarth.leibniz-lsb@tum.de (M.W.); j.benthin.leibniz-lsb@tum.de (J.B.); 2 TUM Graduate School, TUM School of Life Sciences Weihenstephan, Technical University of Munich, Alte Akademie 8, 85354 Freising, Germany; 3 Chair of Nutritional Systems Biology, TUM Junior Fellow, School of Life Sciences, Technical University of Munich, 85354 Freising, Germany

**Keywords:** fava bean protein nanofibrils, atomic force microscopy, Young’s modulus, MCC-13 cells, pH, lipid bilayers, food texture, mechanosensitive ion channels, fat receptors

## Abstract

Functional amyloids (protein nanofibrils, PNF) synthesized from plant sources exhibit unique physicochemical and nanomechanical properties that could improve food texture. While environmental factors affecting PNFs are well-known, scientific evidence on how cells (focus on the oral cavity) respond to them under physiological conditions is lacking. Self-assembled PNFs synthesized from fava bean whole protein isolate show a strong pH- and solvent-dependent morphology and elasticity modification measured by atomic force microscopy (AFM). After incubation of PNFs with an oral mechanosensitive model cell line at pH 7.3, difference in cell-surface roughness without significant changes in the overall cell elasticity were measured. The role of cell membrane composition on supported lipid bilayers was also tested, showing an increase in membrane elasticity with increasing fibril concentration and the possible impact of annular phospholipids in binding. Genetic responses of membrane proteins involved in texture and fat perception were detected at the mRNA level by RT-qPCR assay and both mechano- and chemosensing proteins displayed responses highlighting an interface dependent interaction. The outcomes of this study provide a basis for understanding the changing physicochemical properties of PNFs and their effect on flavor perception by altering mouthfeel and fat properties. This knowledge is important in the development of plant-based texture enhancers for sensory-appealing foods that require consumer acceptance and further promote healthy diets.

## 1. Introduction

There is a growing demand for the adaptation of plant-based food alternatives to meet increasing environmental constraints and to reach sustainable development goals. One critical factor in introducing such alternatives is consumer acceptance through subtle adaptation of existing food characteristics. Food formulations must convey the necessary odor and taste qualities (bitter, sweet, salt, sour, and umami) to achieve the required sensory perception, both driven, primarily, by chemosensation. However, another sensory feature determining food flavor that has been overlooked so far is food texture, i.e., mouthfeel, which is primarily driven by the somatosensory system. In contrast to odor and taste, mouthfeel refers to the everyday mechanical sensations of foods and beverages and depends primarily on their physical properties (e.g., viscosity and elasticity) linked to their chemical composition [1]. Hence, there is a need to design and characterize biomaterials that can provide the desired textural response. Functional amyloids derived from plants (hereafter referred to as protein nanofibrils) are one such biomaterial that has recently attracted attention for its ability to address the above concerns [2,3]. These cross-linked ß-structured fibrils are resistant to proteolytic degradation [4] and have been shown to have no pathological effects and no biological role in different species, including humans [5]. These nanofibrils possess strong nanomechanical properties that are remarkably similar to other fibril sources [6]. To explore the use of these protein nanofibrils (PNFs) for tailor-made applications, knowledge of their behavior in changing microenvironments is crucial, as food formulations are heterogeneous mixtures designed to meet the needs of sensory perception.

Literature indicates that leguminous plants are suitable sources for PNFs and can be synthesized in vitro by exposing plant proteins to an acidic environment at high temperatures [2,7]. Of these, fava beans are of interest as they contain 26–33% protein on a dry weight basis [8] with high concentrations of the amino acids leucine and lysine. Fava bean protein isolate also contains high levels of globulin proteins [9,10] (vicilin and legumin), which can form PNFs and have excellent hydrodynamic and rheological properties [11]. However, similar to a food formulation, the viscoelastic and rheological properties of these PNFs depend on the environment and are influenced by pH, temperature, and incubation time, to name a few [12]. While the industrial applications of PNFs as nanomaterials have been known for some time [13], the use of PNFs as potential texture enhancers while maintaining sensory quality is still in its infancy and requires detailed scrutiny. Previous studies have shown that pH shift induces a morphological change in PNFs [14,15,16], altering its structural properties. However, these studies were carried out by adjusting the pH of the environment in which they were synthesized in (i.e., acidic conditions), which is unsuitable for biological systems. Therefore, characterization of PNFs at the physiological pH of 7.3 in a biological buffer such as HEPES is required. Here, we are investigating PNFs from a biophysical perspective, examining the molecular and cellular responses by measuring the nanomechanical changes, specifically Young’s modulus, at biological membrane interfaces using bioAFM at different pH levels [17,18,19,20].

In this communication, we report and discuss how PNFs might act as a stimulant on a mechanosensitive oral cell line, generating responses without altering the overall cell morphology and elasticity. Furthermore, a gene regulation assay by RT-qPCR enabled us to identify possible membrane proteins involved in PNFs interaction. As the interaction mechanism is interface-dependent, the influence of membrane components (i.e., phospholipids) on PNFs role was studied using a biomimetic system (supported lipid bilayers) and PNFs adsorption was determined in a concentration-dependent manner, along with the speculative mode of binding. We believe that the obtained multifaceted results form a precedent in exploring PNFs as a texture enhancer for mouthfeel sensation while maintaining sensory qualities and consumer acceptance.

## 2. Materials and Methods

### 2.1. Synthesis of Protein Nanofibrils from Fava Bean Protein Isolate

Whole fava bean protein isolate (FPI) was a kind gift from EuroSoy GmbH (Hamburg, Germany). Among the various techniques available for the synthesis of PNFs [13], the heat-induced method was used by adapting the protocol described by Herneke et al. [21]. Briefly, FPI powder was dissolved in 10 mM HCl to obtain a final concentration of 50 mg/mL. After mixing the solution, the sample was centrifuged at 3700× *g* for 15 min at RT. The supernatant was then passed through a 0.45 µm nylon syringe filter, and the solution was dried at 105 °C for 3 h on a hot plate. The dried protein solution was resuspended in 10 mM HCl, and the pH of the solution was adjusted to 2.0. The solution was then incubated at around 65 °C for 18 h on a hot plate, forming the fibrillated sample. The final concentration of the fibrillated sample was estimated using the BSA method and stored at 4 °C.

### 2.2. Physiological Adaptation of PNFs

Since PNFs were synthesized at non-physiological pH (acidic), the solvent and pH were adapted by diluting the PNFs directly in 50 mM HEPES at pH 7.3. It was critical to make sure that there was no gradual shift to the final pH of the solution. Hence, the final volume of PNFs to be added to the buffer was calculated such that it amounted to not more than 5–10% volume percentage compared to the total solution volume. This caused a slight pH shift, but a negligible reduction in the final pH between 6.9 and 7.1, well within the range of pH values in the oral cavity [22]. These fibrils were further used for experiments.

### 2.3. Thioflavin T Assay

Thioflavin T chemically detects the ß-sheets structure of PNFs by binding to it, confirming fibril formation. The fluorescence assay protocol was adapted directly from Herneke et al. [21]. Fluorescence was measured in 96 well plate readers using TECAN Infinite M200 (Maennedorf, Switzerland).

### 2.4. MCC-13 Cell Culture

Merkel cells (MCC-13; CellBank, Sydney, Australia CBA-1338) were cultured at 37 °C and 5.0% CO_2_ in RPMI 1640 (with 2 mM L-Glutamine, 25 mM HEPES, 2.2 g/L NaHCO_3_; Pan Biotech, Aidenbach, Germany) containing 15% FBS and 1% Penicillin and Streptomycin according to the protocol provided by CellBank Australia. Cells were passaged twice a week, after reaching a 70–80% confluence. In brief, passaging was performed by dissociating the cells with trypsin/EDTA (0.05%/0.02% in DPBS without calcium and magnesium, PAN Biotech Germany) for 5 min at 37 °C after removing the media and washing once with 1× phosphate-buffered saline (PBS, without calcium and magnesium, VWR International Belgium). Cells were used after two to 25 passages after thawing, in accordance with Leonard et al. 1995 [23], where the cell line was characterized.

### 2.5. PNFs Interaction with Merkel (MCC-13) Cells

Cells were seeded in dishes (9.2 cm^2^; Techno Plastic Products AG, Trasadingen, Switzerland) 24 h prior to the experiments, at a confluence of 70–80% (300,000–350,000 cells). Right before starting the experiments, cells were washed with PBS to remove dead cells and kept in 2 mL of culture media (RPMI 1640). Live-cell imaging was conducted at physiological conditions (37 °C using temperature controller and 5.0% CO_2_ using CO_2_ Controller 2000, PeCon, Erbach, Germany) on an inverted microscope (Zeiss Axio Observer 7, Oberkochen, Germany with 10x/0.95). Before PNFs incubation, the mechanics of cells were probed by AFM operated in PF-QNM mode. Later, 1 mL of PNFs solution was spiked into the Petri dish by replacing 1 mL of media (total volume is fixed) with required concentrations of 0.025 mg/mL and 0.125 mg/mL maintained. AFM measurements were started after 5 min of incubation to allow the PNFs to settle and finished within 40 min.

### 2.6. Formation of Phospholipid Liposomes

A phospholipid biomimetic system was used to study the interaction between lipid bilayers and PNFs. In brief, 5 mg/mL 20% SoyPC lipids (Avanti Polar Lipids, Alabaster, AL, USA) dissolved in chloroform solution were dried under N_2_ gas and in an Eppendorf vacuum evaporator overnight. The dried lipid film was then resuspended in 50 mM HEPES buffer. Due to heterogeneous phospholipid composition, (i.e., 29% phosphatidylcholine, 30% charged lipids (phosphatidylethanolamine and phosphatidylinositol), and 4% phosphatidic acid), the lipid solution was sonicated for 5 min to attain uniform mixing. The sonicated solution was extruded through a mini-extruder (Avanti) containing a 100 nm polycarbonate membrane to form unilamellar vesicles. The vesicles were stored at 4 °C in an amber glass bottle and used within 3 days of synthesis.

### 2.7. Interaction of PNFs with Supported Lipid Bilayer (SLBs)

100 µL of preformed unilamellar vesicles were incubated with an equal volume of PNFs at different concentrations such that the final phospholipid: PNF ratio was 1:0.005 and 1:0.025. This corresponded to a PNFs concentration of 0.025 mg/mL and 0.125 mg/mL. Each of these samples was incubated for around 30 min, and the treated vesicles were deposited on the surface of freshly cleaved mica for 20 min. The vesicles would then break down to form SLBs due to a process called vesicle fusion. The mica surface was then washed twice with 150 µL buffer to remove any unadsorbed vesicles, and the sample was subjected to AFM measurements.

### 2.8. Atomic Force Microscopy (AFM)

AFM-based measurements and analysis were performed on BioAFM NanoWizard V (Bruker Nano GmbH, Berlin, Germany), coupled to an inverted optical microscope (Zeiss Axio Observer 7) to derive morphological and mechanical data on PNFs. PNFs were deposited on a freshly cleaved atomically flat mica for 10 min and imaged in liquid. Unless specified, all experiments were carried out in 50 mM HEPES buffer at RT. Peak-force Tapping Quantitative nano mechanical imaging, a Force-Distance (FD) mode in AFM, was employed to characterize the mechanics of PNFs. Here, the AFM cantilever oscillates in a sinusoidal manner at a frequency and amplitude well below its peak force resonance frequency, and the sample is contoured pixel by pixel to record an FD curve. Each FD curve provides a multi-parametric output where the height and Young’s modulus (i.e., elasticity) of the sample are obtained. The resolution of each FD-map generated was 256 × 256 pixels. The selection of a suitable substrate for PNFs deposition is crucial for determining accurate Young’s modulus. Here, mica is used as the effect of substrate deformation on Young’s modulus is negligible. Due to its rigid support and very smooth surface (roughness ~0.5 nm), the calculated Young’s modulus and surface roughness are true to the sample. Additionally, as the measurements are done in liquid using soft cantilevers, its high sensitivity detects small forces (e.g., hydration layer on mica) causing the surface stiffness of mica not measured. Hence, it is a suitable support for biomechanical quantifications.

For cell measurements, PFQNM-LC-V2 cantilevers (Bruker) with a nominal tip radius of 70 nm were used to record images of cell surfaces (20–50 μm^2^) and probe the nanomechanics. The pre-calibrated spring constant (nominal value of 0.1 N/m) was used to determine the deflection sensitivity using the thermal noise method [24]. The tip interacted with the cells at an oscillation amplitude of 900 nm and frequency of 625 Hz at an optimal set point of 0.5 nN. For comparative analysis, the same cell was probed before and after treatment with PNFs. This was done by using an optical image overlay with the inverted microscope on which the AFM head is mounted, allowing the coordinates of the cell to be tracked and recorded. This information is then used for further measurements. The AFM measurements were carried out over a period of three different days (confirming three biological replicates) for each PNF concentration tested.

For measuring the mechanics of PNFs and SLB-PNFs interaction, a triangular Si_3_N_4_ cantilever (ScanAsyst Fluid, Bruker) having a nominal tip radius of 20 nm and spring constant of 0.35 N/m was used. The force maps were generated with an oscillation amplitude of 45 nm and frequency of 1.6 kHz at an optimal set point of 1.5 nN. The spring constant of the cantilevers was calibrated before the measurement using the thermal noise method.

### 2.9. Data Analysis

Each pixel of a FD curve generates a force curve (containing approach and retract curve). The approach curve is then extracted and baseline subtracted (to eliminate any noise in the data) followed by contact point identification (between tip and sample). The slope of each curve was calculated and the DMT model (contact mechanics model) was applied to it to determine Young’s modulus. The DMT model was chosen because it takes into account adhesion forces between the tip and the sample, which is important because the interaction of PNFs with cells was being tested for the first time and any potential role of adhesion is unknown, and the deposition of PNFs on the cell membrane would influence the contact mechanics, affecting the modulus data. The model also makes fewer assumptions about the contact geometry with the sample, providing more accurate data for small indentation depths. The Young’s modulus calculations were carried out using Bruker Data Processing software (v 8.0.85). The Young’s modulus obtained is further analyzed in OriginLab (v 9.8.5.212). Height profiles of PNFs were obtained after plane fit and image levelling to identify flatly deposited regions.

### 2.10. Gene Response Data from PNFs-Cells Interaction

Quantitative real-time PCR was conducted on three biological replicates of cells, which were seeded 24 h before incubation in a 6-well plate (9.5 cm^2^; Corning™ Costar™) to obtain a confluence of 70–80% (500,000–600,000 cells). The cells were washed with PBS, treated with either 50 mM HEPES buffer as control or PNFs in 50 mM HEPES buffer (0.05 and 0.25 mg/mL, pH 7.3), and stored in the incubator. After 1 h, the treatment was removed, and cells were again washed with PBS and lysed with lysis buffer containing 2-mercaptoethanol. RNA extraction was conducted with the peqGOLD MicroSpin Total RNA Kit (VWR) according to the manufacturer’s protocol. Lysed cells were then mixed with 70% ethanol and loaded onto the column by centrifugation (13,000 rcf). After one wash with wash buffer and two washes with 80% Ethanol, the RNA was eluted with nuclease-free water. Synthesis of cDNA was performed with the iScript™ gDNA Clear cDNA synthesis kit (Bio-Rad, Feldkirchen, Germany). In this step, the RNA is diluted with nuclease-free water to obtain the same amount of RNA in each sample and mixed with DNase to digest any remaining genomic DNA in the samples. The cDNA is then synthesized by adding reverse transcriptase and incubating it in a thermal cycler (C1000 Touch™ Thermal Cycler, Bio-Rad, Germany). Gene expression analysis of the three biological and two technical replicates per sample was carried out by mixing the primers, synthesized cDNA and Advanced Universal SYBR Green Supermix (Bio-Rad, Germany). The measurement itself was performed by using the CFX96 Touch Real-Time PCR Detection System (Bio-Rad, Germany). To compare the control and the corresponding treated sample, the Cq values were normalized to a Reference Gene Index (RGI) based on the expression of the reference genes GAPDH, ActB and PPIA. Changes in gene expression are described as the fold change of the normalized Cq values of the treated samples relative to the control.

## 3. Results and Discussion

### 3.1. Influence of Microenvironment on Morphology and Elasticity of PNFs

**Figure 1:**
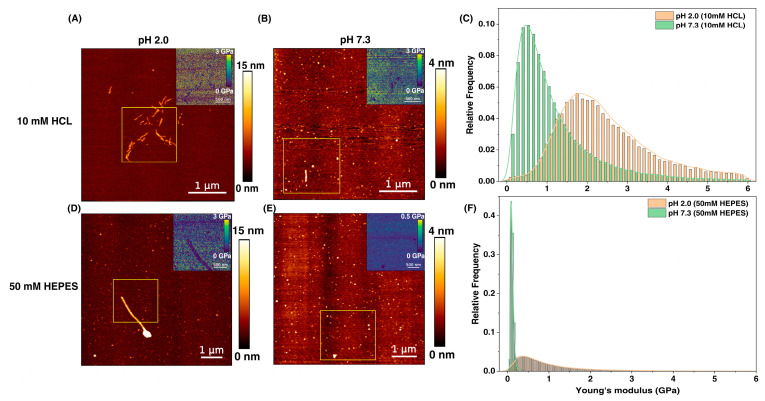
Morphology and elasticity of PNFs measured by bioAFM under different solvent and pH conditions. (**A**) At pH 2.0 in 10 mM HCL, PNFs have a thickness of 3–5 nm and a length of ~500 nm with a mean elasticity of ~2.1 GPa (**C**). The elasticity map is shown in the inset (**A**). When pH was shifted to 7.3, loss of fibril structures was visible (**B**), indicating remodeling of PNFs with a mean elasticity reduced to ~0.5 GPa (**C**). In 50 mM HEPES buffer, pH 2.0 (**D**), few elongated fibrils were observed but with more pronounced structural aggregates and a mean elasticity of ~0.5 GPa (**F**). At pH 7.3 (**E**), the PNFs displayed only circular aggregates entirely deviating from their original structure, with the mean elasticity further reduced to ~0.1 GPa (**F**). The yellow square box in each figure is a representative area for the elasticity map. The histograms (**C**,**F**) represent only the specific interaction, i.e., the interaction between the AFM tip and PNFs. For simplicity, non-specific interactions (i.e., AFM tip with mica) are not shown here, and the full histogram is available for reference in Figure S3A. The range for Young’s modulus (i.e., 6 GPa as observed in the histogram) was chosen keeping PNFs synthesized at pH 2.0, 10 mM HCL as reference. For better visualization of the elasticity map, the legend has been adapted accordingly. Scale bar (inset) = 500 nm. The histograms were fitted with Kernel density estimation for mean values. Sample force curve generated is shown in Figure S2A.

Upon synthesizing PNFs from whole fava bean isolate (FPI) at pH 2.0 in 10 mM HCL, bioAFM imaging revealed the self-assembly of fibrils with a thickness of 3–5 nm and a length of ~0.5 µm, as shown in Figure 1A. The morphology obtained is in agreement with the literature [21]. The presence of ß-sheet rich structures was also confirmed using Thioflavin T assay [25], a well-known in vitro fibril indicator (Figure S1A). Peak force quantitative nanomechanical (PF-QNM) imaging was used to estimate Young’s modulus of the PNFs, which was ~2.1 GPa, as shown in the data distribution of Figure 1C. The extraction of elasticity data from force curves is shown in Figure S2. The elasticity obtained was almost 1.5 times lower than that of ß-lactoglobulin PNFs formed from whey protein [6] indicating the formation of softer fibrils. When pH was shifted to 7.3, the PNFs showed a structural transition to short circular structures/aggregates, suggesting remodeling of the fibrils (Figure 1B). While their thickness was maintained, the elasticity was drastically reduced to ~0.5 GPa (Figure 1C). Of note, the nature and kinetics of the formed fibrils (i.e., oligomers, protofilaments or mature fibrils) were not characterized, as this is beyond the scope of this article, but could have a definite role in influencing fibril elasticity (e.g., mature fibrils can undergo secondary nucleation [26] in forming further aggregates). To test if the pH-induced fibril reorientations resulted in a reduction in the total number of ß-sheets leading to secondary structure modifications, a Thioflavin T assay was performed. This indicator showed no overall decrease in the number of ß-sheets (Figure S1B).

Next, we changed the PNFs solvent from 10 mM HCL to 50 mM HEPES, a commonly used biological buffer known for maintaining pH with little interference from other biomolecules such as metal ions. Upon analysis, it was found that the characteristics of PNFs in 50 mM HEPES, pH 2.0, did not resemble anywhere close to PNFs in an HCL environment, showing the presence of few elongated fibrils but pronounced aggregates (Figure 1D). The presence of HEPES caused a considerable shift in elasticity with mean Young’s modulus values of ~0.5 GPa at pH 2.0 and further decreasing to ~0.1 GPa (Figure 1F) at pH 7.3. Morphologically, fibrils at 50 mM HEPES, pH 7.3 (Figure 1E) resembled PNFs in 10 mM HCL, pH 7.3 (Figure 1B), demonstrating that the final microenvironment determines the fate of fibril properties with intermediate fibrillation stages and various other transition states of minimal significance [27].

### 3.2. Impact of PNFs on Quantitative Mechanics of MCC-13 Cells

**Figure 2:**
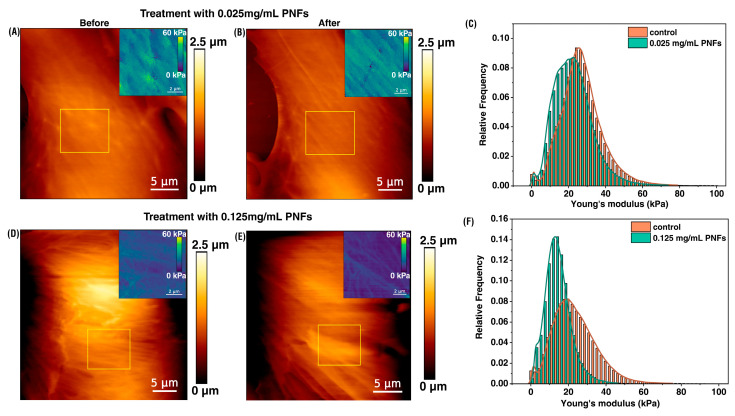
In-situ live-cell nanomechanical probing of MCC-13 model cells before and after PNFs treatment. Morphologically, the cell surface showed no visible changes before (**A**,**D**) and after (**B**,**E**) PNFs treatment, at different concentrations. The overall cell elasticity was also remarkably unchanged (**C**,**F**), with a slight variation in cell rigidity from ~25 kPa (control) to ~22 kPa at low PNF concentration. A similar shift in Young’s modulus was detected at high PNF concentration (**F**), with a slight decrease in elasticity from ~20 kPa (control) to ~15 kPa. The inset shows a representative elasticity map of the yellow squared region obtained during measurements. Scale bar (inset) = 2 µm. Figure S2B shows a representative force curve obtained during measurements and Figure S5 shows the overlay of the optical image and the AFM scanned area of MCC-13 cells. The complete histogram of cell elasticity is shown and was fitted with Kernel density estimation for mean values. A total number of nine cells per condition were analyzed.

Following the physicochemical characterization of PNFs, experiments were continued at pH 7.3, 50 mM HEPES to explore the cellular nanomechanical response elicited by these fibrils in physiological environment. The Merkel carcinoma cell (MCC-13) line is a well-established model cell line for transducing biological signals due to touch sensation [28,29]. We believe that the physical attributes of PNFs promoted by their fibrous alignment and changing aspect ratio could induce a similar action through potential interactions with the cell surface, influencing the overall interfacial dynamics and rheological properties [3]. The selection of MCC-13 cells was also based on the criteria that these specialized cells are present on the skin’s surface [30], including the oral cavity, and contain an increased number of mechanosensitive protein channels (i.e., Piezo1/2) [31], which have been proposed to be involved in oral texture perception [1], as well as making synaptic contacts [32,33]. Two concentrations (0.025 and 0.125 mg/mL) of PNFs were chosen based on previous toxicity studies, showing their safe consumption for humans [4]. When these cells were treated with 0.025 mg/mL PNF for 30 min, the cell’s elasticity slightly changed from ~25 kPa (control, before, Figure 2A) to ~22 kPa (after, Figure 2B), indicating a slight variation in the cell’s rigidity (Figure 2C). When the PNF concentration was increased 5-fold to 0.125 mg/mL, Young’s modulus was observed with a mean elasticity of ~15 kPa compared to the control, i.e., ~20 kPa (Figure 2D–F). The histograms show the cumulative data from nine cells per condition, and while there is a decreasing trend in cell elasticity, the changes cannot be characterized as statistically significant. Whilst one could speculate about insufficient adsorption of PNFs on the cell surface based on incubation time, it is also possible that increased PNFs-PNFs interactions lead to the formation of self-aggregates [34], which hinder their binding to the cell surface. As it was difficult at this stage to determine any preferential contact, we deduced potential PNF interaction with the cell surface in terms of cell-surface roughness (Rq), which is a physical quantity proven to be a crucial parameter in molecular interactions influencing cellular functions [35,36]. To confirm whether PNF’s viscoelastic properties could trigger such changes, we calculated the RMS value of Rq and found that with nanofibril treatment, the Rq of MCC-13 cells reduced around 30% with respect to control at low PNF concentration (Rq = 194.12 ± 64.11 nm; control: Rq = 278.85 ± 30.85 nm) and around 24% at high PNF concentration (Rq = 232.85 ± 53.60 nm; control: Rq = 305.89 ± 76.81 nm). These reduced Rq indicate significant changes to the cell membrane integrity and function, which could support molecular adhesion [37,38].

To understand membrane modulations from the perspective of PNFs, the protein composition of fava beans is crucial. Vicilin and legumin, two well-known globulins, play an important role in fibril formation. Their denaturation, which occurs during thermal or pH-dependent processing, changes their physicochemical attributes and exposes hydrophobic amino acids, altering the surface hydrophobicity of PNFs [39]. This in turn may affect their molecular interactions with the cell membrane. However, this requires a detailed analysis, beyond the scope of the article. Additionally, globulins are more soluble at higher pH levels, such as in foaming applications, where amphiphilic molecules reduce surface tension and improve the functional properties of proteins [11]. Phospholipids on the cell membrane could facilitate this role in helping to stabilize PNF interactions. The increased reduction in Rq can also be attributed to the morphology of PNFs as it was shown on neuroblastoma cells that fibril’s oligomeric aggregates (as seen in Figure 1E) decrease the Rq more favorably than fibrillar structures [40]. Single cells without crowding or overlap were only considered for Rq estimation. Based on the above results, we tested two scenarios to elucidate the interaction of PNFs with the cells. Firstly, we probed the interaction between phospholipids and PNFs using membrane mimetic systems (supported lipid bilayers) to resolve the interface dependence and adhesion of PNFs. Secondly, a gene regulation assay using RT-qPCR was performed on MCC-13 cells after incubation with PNFs to check for stimulation of membrane protein activity at the mRNA level.

### 3.3. Supported Lipid Bilayer (SLBs)-PNFs Interaction

**Figure 3:**
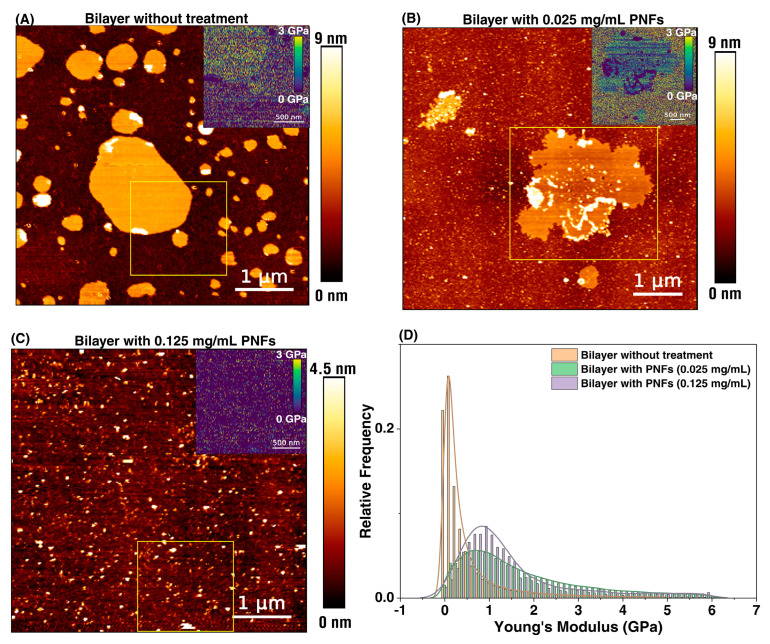
Liquid AFM imaging showing height and elasticity profiles of supported lipid bilayers after PNF interaction. Before treatment, control bilayers formed lipid patches with an average height of ~4.5 nm (**A**) and Young’s modulus of ~0.1 GPa ((**A**) inset, and (**D**)). The presence of charged phospholipids limits the formation of a continuous bilayer covering the entire mica surface when using the vesicle fusion method. At low PNF concentration, adsorption of the fibrils on the surface of the bilayer was detected (**B**), increasing the overall elasticity and thereby raising membrane rigidity (inset in (**B**,**D**)). PNFs also unveiled distinct edge-based binding with the lipid bilayer at low concentrations ((**B**), Figure S4A). At high PNF concentrations, detecting the presence of an intact lipid bilayer was more difficult (**C**), indicating disintegrated membrane structures ((**C**), Figure S4B). However, an increase in Young’s modulus was measured for the formed, smaller structures (**D**). Similar to Figure 1C,F, only the specific interactions are presented, i.e., the interaction between the AFM tip and PNF-bilayer. The full histogram is available for reference in Figure S3B. The inset shows a representative elasticity map of the yellow squared region. Scale bar (inset) = 500 nm. The histograms were fitted with Kernel density estimation for mean values. Three replicates of the bilayer patches were analyzed per condition. Figure S2C shows a representative force curve.

Heterogeneous SLBs from soy lipid extract [41,42,43] were formed on the surface of mica to mimic mammalian cell composition. The deposition of lipid vesicles on a hydrophilic surface (mica) results in the formation of lipid bilayers (Figure 3A) corresponding to a height of 4–5 nm [43,44] as observed on a native cell membrane. Treatment with PNFs at a low concentration (0.025 mg/mL) resulted in the adsorption of fibrils onto the surface of the lipid bilayer visible as surface aggregates (Figure 3B). The total height of the lipid bilayer was found to be highly variable (up to 20 nm) and appeared to depend solely on fibril deposition when compared to the control bilayer. Apart from direct deposition, a distinct behavior of PNFs interaction was also observed. The periphery of the lipid bilayer displayed kinks, which we believe could be the initial adhesion sites for fibril binding, progressing to overall deposition (Figure S4A). However, the binding should be dependent on the lipid composition. Literature suggests that mature fibrils often have a lower affinity to lipid bilayers compared to fibrils in their early stages [45]. However, those that do bind interact by targeting the lipids in the annulus region with possible uptake. If this is the case, the presence of kinks and cracks observed at the edges of the bilayer could explain the likely mode of molecular interaction and the changes in the detected lipid membrane elasticity, as shown in Figure 3D. The surface adsorption of fibrils resulted in an increase in membrane elasticity from ~0.1 GPa (control) to ~0.8 GPa, further highlighting the role of phospholipids in modulating membrane integrity and functionality. At high PNF concentrations (0.125 mg/mL), it was difficult to detect the presence of an intact lipid bilayer. Instead, a mostly disintegrated morphology was observed (Figure 3C). The absence of an intact bilayer on the mica surface (Figure S4B) also confirmed the possibility of lipid uptake by the PNFs, with Young’s modulus values further increasing to ~1 GPa (Figure 3D).

The results discussed above could be indicative of PNFs-induced defects on cell membranes causing toxicity. However, as mentioned earlier, the PNF concentration tested here is within the reported values (i.e., 0.25 mg/mL) where no toxicity was observed [4], and we also did not observe any loss of cell viability during the AFM live cell experiments (Figure S5). Based on the above, it is reasonable to assume that the detected membrane perturbations are necessary modulations induced by the PNFs to generate a response for subsequent downstream signaling. While the results confirm that phospholipids facilitate the interaction of PNFs, unlike the cell membrane, SLBs are not a continuous bilayer system but rather islands of lipid patches, which could also explain the limitation of the mimetic model and the effects of fibrils on it.

### 3.4. Gene Response from MCC-13 Cells

**Figure 4:**
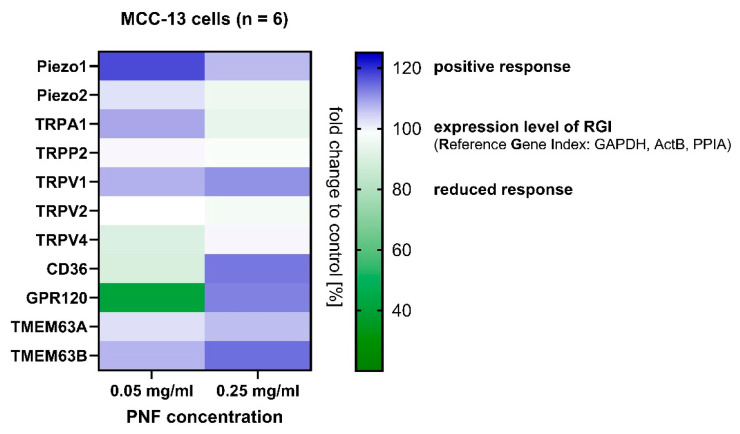
RT-qPCR gene regulation assay of protein response at mRNA level in MCC-13 cells after PNF treatment. Mechanosensitive ion channel Piezo1 showed a positive response at low PNF concentration compared to high PNF concentration. Other mechanosensitive receptors, i.e., TMEM63B, revealed a concentration-dependent increase in gene response. Fat receptors CD36 and GPR120 had a reduced response at low PNF concentrations but increased to a positive response at high PNF concentrations, indicating the contribution of fibril characteristics in regulating mechanosensors and chemosensors. Other mechanosensitive ion channels such as TRP channels displayed selective behavior, i.e., only TRPV1 showed a concentration-dependent positive response, whereas TRPA1 did so only at low concentrations. Gene responses were quantified based on the reference gene index set at 100 (fold change in percentage), and values for proteins were obtained correspondingly. Here n = 6 (three biological replicates with two technical replicates each).

With the changes in Rq indicating cell surface modifications and PNFs displaying interfacial dynamics from bilayer studies, we wanted to further understand whether membrane proteins show behavioral responses upon PNF interaction. Our focus in this regard was more towards mechanosensitive (MS) proteins (e.g., Piezo1/2) as they respond to stimulants involved in touch sensation as mentioned earlier. Also, a recent generic mathematical model in texture perception has highlighted that MS channels generate action potential in response to changes in surface roughness and dynamic sliding [46]. Therefore, the mRNA levels of human MS ion channels proposed to be involved in mouthfeel were measured by RT-qPCR in MCC-13 cells after PNF treatment (0.05 and 0.25 mg/mL, 1 h) and expressed as fold change (FC in %). Table S1 provides details on the primer sequences of interest and their melting temperatures. To ensure a sufficient signal in RNA levels, the concentration of fibrils was increased while maintaining the 5-fold difference. The screening (Figure 4) revealed that, in comparison to untreated control cells (FC = 100), Piezo1 protein showed an increased positive response at low PNF concentration (0.05 mg/mL, 117.5 ± 1.2), compared to high PNF concentration (0.25 mg/mL, 106.8 ± 1.3). A negligible response was detected for the Piezo2 protein at both concentrations (103.0 ± 1.2 and 95.3 ± 1.1). Similar to Piezo1, a genetic response was measured for the TMEM63(A/B) [47,48] genes, which are mechanosensors involved in calcium transport. TMEM63B showed a significant response to high PNF concentration (114.2 ± 1.2), compared to low PNF concentration (107.3 ± 1.2), while TMEM63A response did not increase for either concentrations (103.1 ± 1.2 and 106.4 ± 1.2).

Since fibrils showed physical adsorption to the cell membrane, affecting membrane elasticity (Figure 3), we additionally investigated whether any other type of membrane protein showed changing RNA levels (e.g., fat/lipid receptors). This is of particular interest as the functionality of transmembrane proteins in general depends on cell membrane constituents, and PNFs themselves are amphiphilic polymers with viscoelastic properties that can promote hydrophobic interactions. Recent literature on catfish fins had shown that adipose tissue contributes to mechanosensitivity [49], and the discovery of lipids embedded in the structures of Piezo proteins or two-pore potassium channel (K2Ps) proteins [50] highlights possible interplay. When examined, we found that long-chain fatty acid receptor CD36 [51] and free fatty acid receptor GPR120 [52] showed a positive response at high PNF concentration (113.4 ± 1.9 and 112.4 ± 1.5, respectively), suggesting a possible role of PNFs in mechanosensation and fat perception. Like Piezo proteins, PNFs influenced RNA levels of similar mechanosensitive ion channels, i.e., TRP channels [53] (TRPV1/V2/V4 and TRPA1), which are known to be altered by perturbations in membrane stretch, curvature, and tension, as well as by external lipids. However, the responses generated were selective, and only TRPV1 showed a positive response at both PNFs concentrations (low: 107.7 ± 1.2, high: 110.7 ± 1.5) whereas TRPA1 only at low PNFs concentration (108.7 ± 1.2). The observed FC highlighted serves as an initial screening in identifying PNF-induced protein responses. Further experiments at the cellular level are needed to confirm the functionality of said protein expression and to study their behavior in the presence of PNFs.

## 4. Conclusions

Protein nanofibrils are polymeric-ordered structures that exhibit discrete rheological properties and could contribute to enhancing food texture [54]. They have been explored for some time for specific applications such as delivery of bioactive compounds [55], development as food functional materials i.e., hydrogels, emulsions [56] or as nutrient enhancers by using them as scaffolds [57]. As in each of the above scenarios, fibrils show structural and functional modifications depending on the external environment; there has been a lack of scientific evidence on the adaption of plant-based protein fibrils at physiological pH. Here we report the first results of understanding the inherent properties of fibrils at different pH using Young’s modulus and morphological parameters. After characterizing the PNFs at the nanometer scale by AFM, we found that the above tested non-toxic PNF concentrations did not alter the overall cell elasticity and morphology of MCC-13 cells but changed their surface roughness suggesting PNF interactions (like deposition). Since the interaction of PNF with cells appeared to be viscoelastic and no significant changes in overall cell elasticity were observed, the question arises as to whether the viscous component of PNF plays a greater role in inducing a cellular response than the elastic component. However, this relationship needs to be investigated in detail. The contribution of membrane components in PNFs interaction was determined using membrane mimetic systems which showed concentration-dependent binding of phospholipids, highlighting their potential functional role in the activity of the protein, with annular lipids contributing to fibril binding. PNFs also elicited gene responses (tested at mRNA level) from both mechano- and chemosensing proteins known or suspected to be involved in oral texture perception, indicating possible cellular responses. The compilation of such biophysical and biochemical data suggests the predominant effect of mechanical forces shaping the behavior of cells and provides evidence for cross-talk between mechanotransduction and biological processes [58]. However, the synthesis of fibrils has some limitations. The current method involves the thermal processing of fava beans, which though can improve digestibility and bioavailability, may result in nutrient loss. One way to overcome this is to reduce the temperatures below the denaturation temperatures of globulins (legumins denature at 95.4 and vicilins at 83.8 °C) [59,60].

The outcomes of this study provide a basis for understanding the changing physicochemical properties of PNFs at physiological pH, as this may affect flavor perception by altering mouthfeel and fat properties. This study attempts to address some of the scientific concerns in the implementation of plant-based nanofibrils as texture enhancers. In addition, it explores the potential of leveraging AFM as a non-invasive tool to investigate the mechanical properties of nanofibrils and cell systems. This knowledge will be important in the development of sensory-appealing foods that require high levels of consumer acceptance and further promote healthy diets.

## Data Availability

The original contributions presented in this study are included in the article/Supplementary Materials. Further inquiries can be directed to the corresponding author.

## Supplementary Materials

The following supporting information can be downloaded at: https://www.mdpi.com/article/10.3390/foods13213411/s1, Figure S1: Thioflavin T assay to detect the presence of ß-sheets in synthesized protein nanofibrils; Figure S2: Sample force-distance (FD) curve and extraction of elasticity (Young’s modulus) data; Figure S3: Full histogram of elasticity data (Young’s modulus) obtained when AFM tip interacted with the samples; Figure S4: AFM images showing PNF deposition on lipid bilayer; Figure S5: Optical image of MCC-13 cells; Table S1: List of primer sequences and its characteristics.
