# Supplementary material for: Fava Bean Protein Nanofibrils Modulate Cell Membrane Interfaces for Biomolecular Interactions as Unveiled by Atomic Force Microscopy
Source: Foods. 2024 Oct 26;13(21):3411. doi: 10.3390/foods13213411 (PMC11545818; doi:10.3390/foods13213411)
Supplement: Supplementary file 1 [file foods-13-03411-s001.zip › foods-3175671-supplementary.pdf]

Supplementary Information

Fava Bean Protein Nanofibrils Modulate Cell Membrane Interfaces for Biomolecular Interactions as Unveiled by Atomic Force Microscopy

Sanjai Karanth[1], Marina Wiesenfarth[1,2], Julia Benthin[1,2] and Melanie Koehler[1,3,*]

[1] Leibniz Institute for Food Systems Biology at the Technical University of Munich, Lise-Meitner-Str. 34, 85354 Freising, Germany

[2] TUM Graduate School, TUM School of Life Sciences Weihenstephan, Technical University of Munich, Alte Akademie 8, 85354 Freising, Germany

[3]Nutritional Systems Biology, Technical University of Munich, 85354 Freising, Germany

*Corresponding author: m.koehler.leibniz-lsb@tum.de

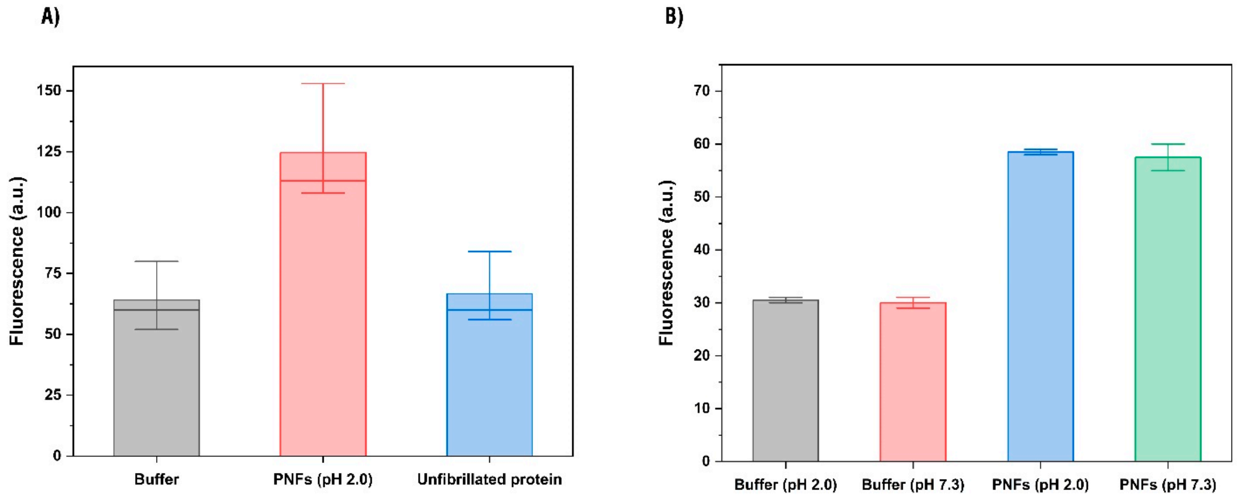

Figure S1: Thioflavin T assay to detect the presence of ß-sheets in synthesized protein nanofibrils. A) At pH 2.0, 10 mM HCL, an increase in fluorescence was observed in comparison with unfibrillated protein, indicating the formation of protein nanofibrils with ß-sheets as a measure. B) With shift in pH to 7.3, no considerable change in fluorescent signal was detected, though morphological change was visible by AFM. This confirms molecular remodeling of PNFs without loss of ß-sheets. Buffer is 10 mM HCL at pH 2.0 and 50 mM HEPES at pH 7.3.

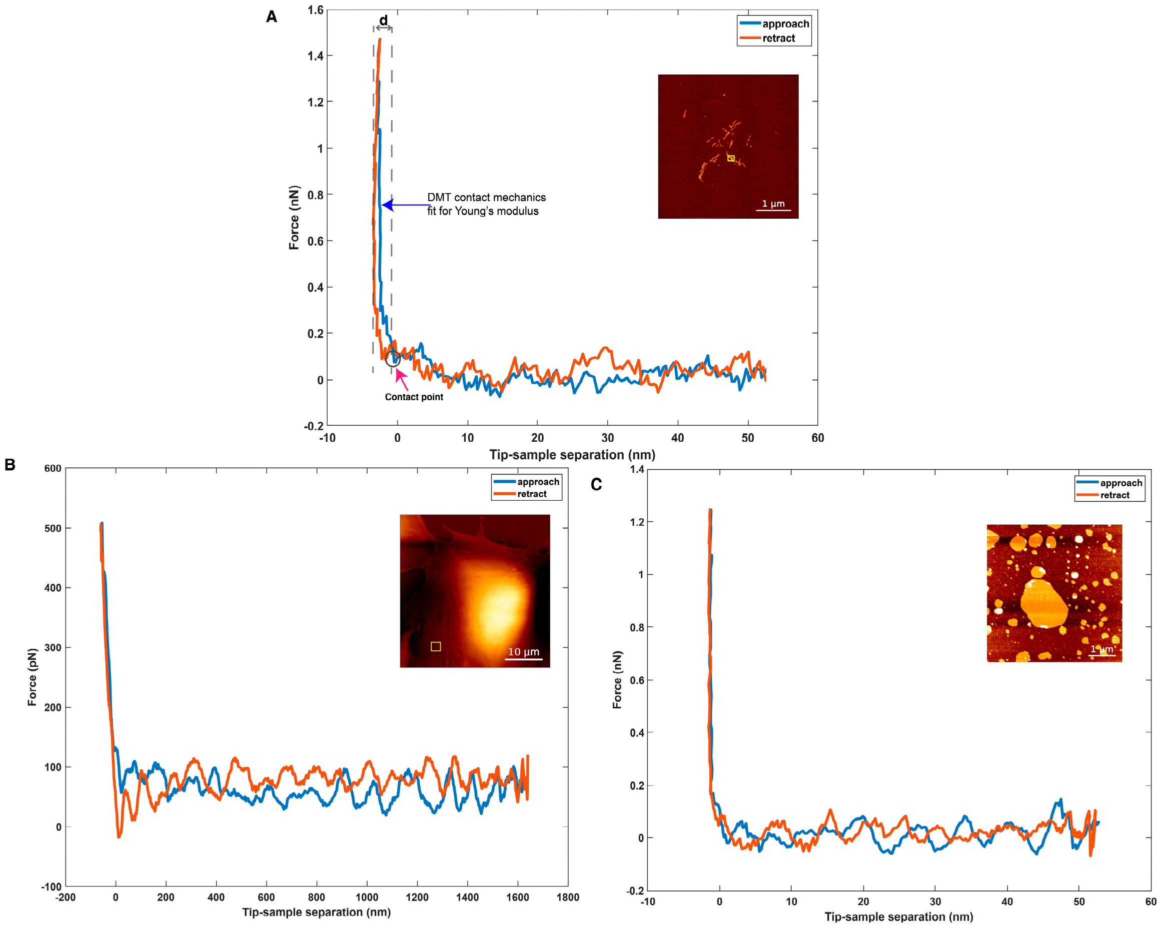

Figure S2: Sample force-distance (FD) curve and extraction of elasticity (Young's modulus) data. A) Inset: AFM image of PNFs at pH 2.0, 10 mM HCL is shown and yellow dot represents the region of the force curve. As the AFM tip moves towards the surface of fibrils and makes contact with the sample at contact point (circled region) approach curve is generated at defined force (called set point). The slope from this approach curve obtained is then fitted with Derjagin, Muller, Toropov (DMT) contact mechanics model to calculate Young's modulus. When the AFM tip is in-contact with the fibril, sample surface indentation is possible and the depth (d) can be measured as shown. Here, the indendation depth of ~1.6 nm. B) Sample FD curve for PNF-cell interaction for high PNF concentration with indentation depth of ~60 nm and C) sample FD curve for control lipid bilayer with indentation depth of ~0.5 nm The region from which the force curves are selected are shown as yellow squares.

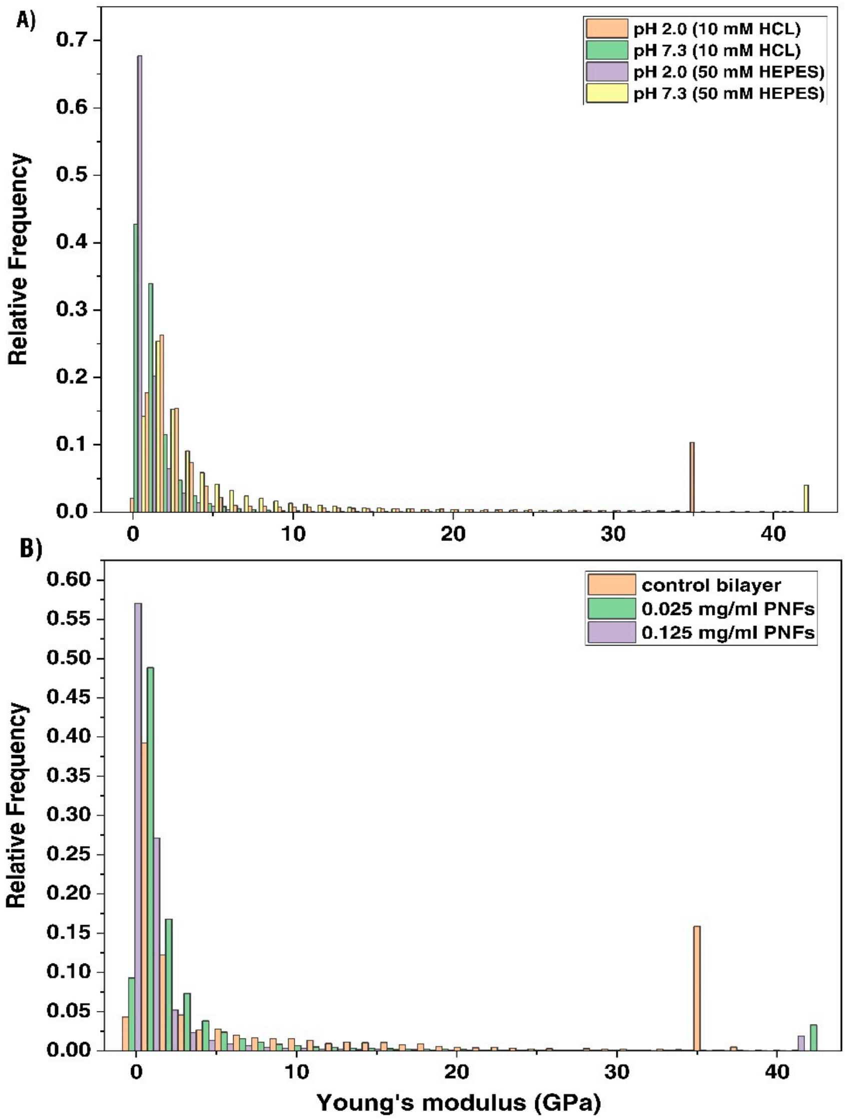

Figure S3: Full histogram of elasticity data (Young's modulus) obtained when AFM tip interacted with the samples. During AFM probing experiments, specific (between AFM tip and PNFs), as well as non-specific (between AFM tip and mica) interactions occur. A) Histogram obtained for samples with pH and solvent variation, and B) shows lipid bilayer properties post PNF interaction. The very high Young's modulus, visible as a single peak at around ~35 GPa can be attributed to non-specific interactions between the AFM tip and mica.

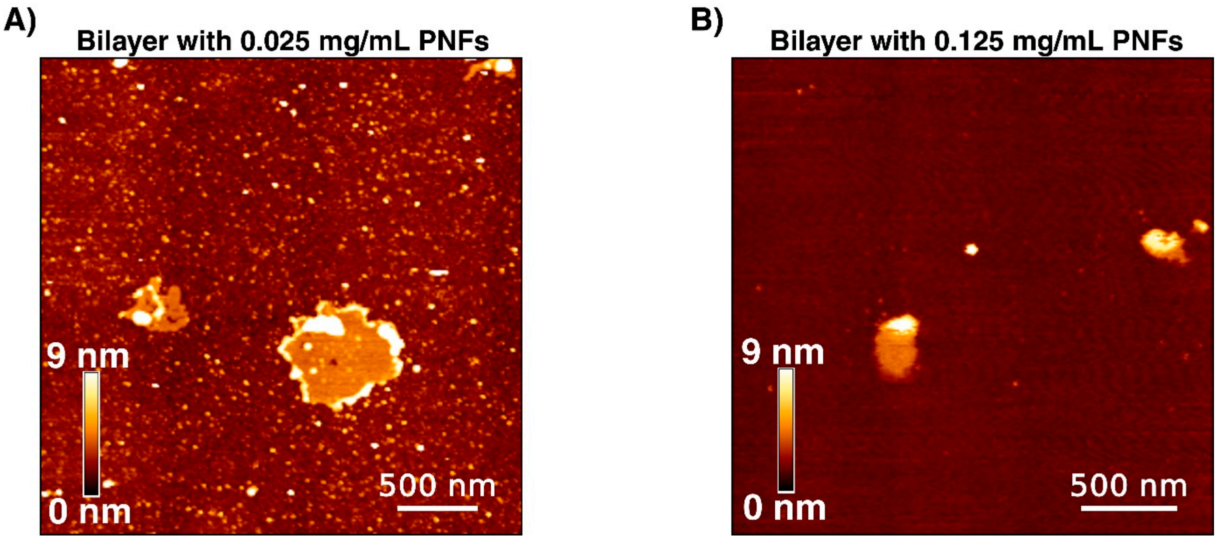

Figure S4: AFM images showing PNF deposition on lipid bilayer. At low PNF concentration, along with deposition of fibrils on membrane (visible as white aggregates), formation of kinks at the bilayer periphery was recognized (A) with possible contribution of annular lipids in binding of PNFs to the bilayer. At high PNF concentration (B) the identification of intact lipid bilayers on the mica was difficult.

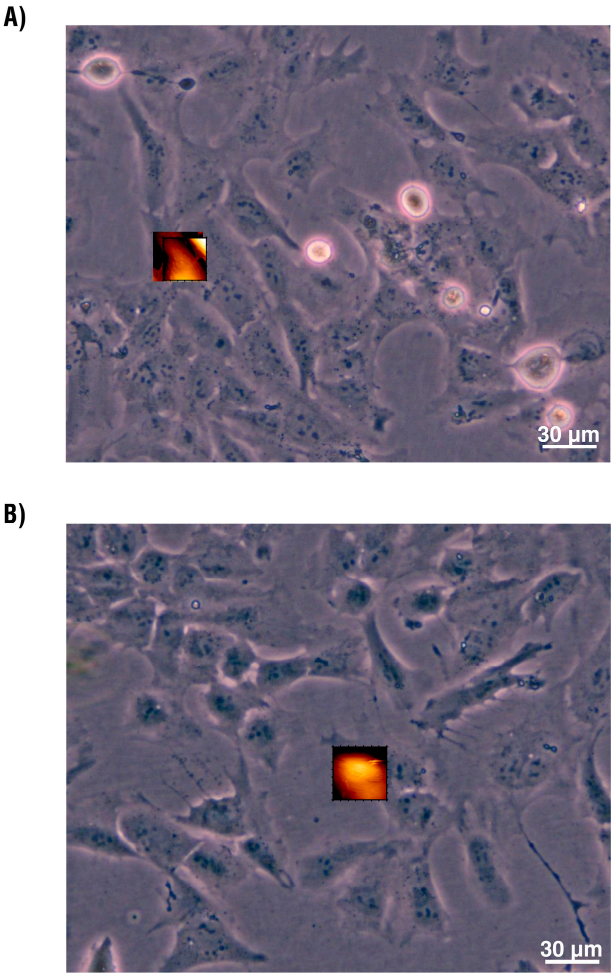

Figure S5: Optical image of MCC-13 cells. The ROI for AFM nanomechanical imaging before and after treatment of PNFs is shown here, i.e., for 0.025 mg/mL (A) and 0.125 mg/mL (B). The viability of cells during measurements was also observed in parallel and indicated no significant reduction.

Table S1: List of primer sequences and its characteristics. Sequences from Bio-Rad are confidential and therefore not online accessible or shared when ordering. The primers are validated by Bio-Rad Laboratories and the specific Assay ID with validation data is shown on the company website.

**Primer**	**Item number/ assay ID (manufacturer)**	**Sequence**	**Melting temp.**	**Further references**
FAM38A (Piezo1) Human qPCR Primer Pair	NM_001142864 (Origene)	forward: CCTGGAGAAGACTGACGGCTAC reverse: ATGCTCCTTGGATGGTGAGTCC	86.0°C	
FAM38B (Piezo2) Human qPCR Primer Pair	NM_022068 (Origene)	forward: GACGGACACAACTTTGAGCCTG reverse: CTGGCTTTGTTGGGCACTCATTG	85.0°C	
TRPA1	customized (Eurofins genomics)	forward: GTTTGGTGAAGAGCATGGGTAC reverse: GTGCACCTTCCCTTCTCCAC	83.0°C	Andersen et al. Mol. Nutr. Food Res. 2023, 67, 2200434 (supplement)
TRPP2	customized (Eurofins genomics)	forward: CTGCGAGGTCTCTGGGGAAC reverse: CGTAGGTCAAGATGCACAAG	81.0°C	Andersen et al. Mol. Nutr. Food Res. 2023, 67, 2200434 (supplement)
TRPV1	customized (Eurofins genomics)	forward: GTTCAAAGACCCTGAGACAG reverse: GTGCTGTCTGGCCCTTGTAG	87.0°C	Andersen et al. Mol. Nutr. Food Res. 2023, 67, 2200434 (supplement)
TRPV2	customized (Eurofins genomics)	forward: GCTGCAGAAAGCCATCTCTG reverse: CTCCTCCACCCTGAAGCAC	86.5°C	Andersen et al. Mol. Nutr. Food Res. 2023, 67, 2200434 (supplement)
TRPV4	customized (Eurofins genomics)	forward: GGCCTATCCTCTTTGACATCG reverse: CTTGGGCAGGCAGGTCTTC	86.5°C	Andersen et al. Mol. Nutr. Food Res. 2023, 67, 2200434 (supplement)
PrimePCR™ SYBR® Green Assay: CD36, Human	qHsaCID0011828 (BioRad)	not accessible	78.5°C	CD36 - PCR Primer Pair - SYBR | PrimePCR | Bio-Rad (Link)
PrimePCR™ SYBR® Green Assay: O3FAR1 (GRP120), Human	qHsaCID0008234 (BioRad)	not accessible	86.0°C	O3FAR1 - PCR Primer Pair - SYBR | PrimePCR | Bio-Rad (Link)
PrimePCR™ SYBR® Green Assay: TMEM63A, Human	qHsaCID0014732 (BioRad)	not accessible	79.5°C	TMEM63A - PCR Primer Pair - SYBR | PrimePCR | Bio-Rad (Link)
PrimePCR™ SYBR® Green Assay: TMEM63B, Human	qHsaCED0003861 (BioRad)	not accessible	84.0°C	TMEM63B - PCR Primer Pair - SYBR | PrimePCR | Bio-Rad (Link)
PrimePCR™ SYBR® Green Assay: PPIA, Human	qHsaCED0038620 (BioRad)	not accessible	84.0°C	PPIA - PCR Primer Pair - SYBR | PrimePCR | Bio-Rad (Link)
PrimePCR™ SYBR® Green Assay: ACTB, Human	qHsaCED0036269 (BioRad)	not accessible	82.0°C	ACTB - PCR Primer Pair - SYBR | PrimePCR | Bio-Rad (Link)

PrimePCR™ SYBR® Green Assay: GAPDH, Human	qHsaCED0038674 (BioRad)	not accessible	86.0°C	GAPDH - PCR Primer Pair - SYBR | PrimePCR | Bio-Rad (Link)
